# Therapeutic Challenges for Cisplatin-Resistant Ovarian Germ Cell Tumors

**DOI:** 10.3390/cancers11101584

**Published:** 2019-10-17

**Authors:** Ugo De Giorgi, Chiara Casadei, Alice Bergamini, Laura Attademo, Gennaro Cormio, Domenica Lorusso, Sandro Pignata, Giorgia Mangili

**Affiliations:** 1 Department of Medical Oncology and Hematology, Istituto Scientifico Romagnolo per lo Studio e la Cura dei Tumori (IRST) IRCCS, 47014 Meldola, Italy; chiara.casadei@irst.emr.it; 2 Department of Obstetrics and Gynaecology, San Raffaele Scientific Institute, 20132 Milan, Italy; bergamini.alice@hsr.it (A.B.); mangili.giorgia@hsr.it (G.M.); 3 Department of Urology and Gynecology, Istituto Nazionale Tumori IRCCS Fondazione G. Pascale, 80138 Naples, Italy; l.attademo@istitutotumori.na.it (L.A.); s.pignata@istitutotumori.na.it (S.P.); 4 Gynecologic Oncology Unit, IRCCS Istituto Oncologico Giovanni Paolo II, 70124 Bari, Italy; gennaro.cormio@uniba.it; 5 Gynecologic Oncology Unit, Department of Woman, Child Health and Public Health, Fondazione Policlinico Universitario Agostino Gemelli, IRCCS, 00168 Rome, Italy; domenica.lorusso@policlinicogemelli.it

**Keywords:** female patients, germ cell tumors, ovarian germ cell tumors, salvage therapy, refractory, high-dose chemotherapy

## Abstract

The majority of patients with advanced ovarian germ cell cancer are treated by cisplatin-based chemotherapy. Despite adequate first-line treatment, nearly one third of patients relapse and almost half develop cisplatin-resistant disease, which is often fatal. The treatment of cisplatin-resistant disease is challenging and prognosis remains poor. There are limited data on the efficacy of specific chemotherapeutic regimens, high-dose chemotherapy with autologous progenitor cell support and targeted therapies. The inclusion of patients in clinical trials is strongly recommended, especially in clinical trials on the most frequent male germ cell tumors, to offer wider therapeutic opportunities. Here, we provide an overview of current and potential new treatment options including combination chemotherapy, high-dose chemotherapy and molecular targeted therapies, for patients with cisplatin-resistant ovarian germ cell tumors.

## 1. Introduction

The European age-standardized incidence rate for female germ cell tumors (GCTs) is 0.4 per 100,000 persons per year [1], whereas that of males is 15-fold higher [2]. GCTs derive from embryonic germ cells, which, instead of differentiating correctly, undergo malignant transformation [3]. They arise in the gonads in >90% of cases. The remaining 10% are extragonadal tumors occurring in the mediastinum, retroperitoneum and, less frequently, central nervous system sites such as neurohypophysis and the pineal gland [4]. This distribution reflects the original route followed by germ cell precursors during embryologic development.

Malignant ovarian GCTs (MOGCTs) are subdivided into dysgerminomatous tumors (the most frequent type) and non-dysgerminomatous tumors. The World Health Organization (WHO) classification of MOGCT is shown in Table 1 [5]. Dysgerminomas express features typical of primordial germ cells and may be associated with dysgenetic gonads and sexual development including the Turner syndrome, testicular feminization and triple X syndrome [6,7]. Most dysgerminomas produce lactic dehydrogenase (LDH) and β-human chorionic gonadotropin (β-hCG), as shown in Table 2 [8]. In gonadal dysgenesis, dysgerminoma derives from a gonadoblastoma, and rarely from an intra-abdominal testis. The majority of these tumors have high mitotic activity [9].

Non-dysgerminoma tumors have multiple histological subtypes that resemble the differentiation occurring during human development. In particular, hallmarks of embryonic differentiation occur in embryonal carcinoma (EC) and teratoma (both mature and immature), while features of extraembryonic differentiations are present in yolk sac tumors and choriocarcinomas [10]. ECs, choriocarcinomas and malignant struma ovarii tumors represent the rarest forms of MOGCTs. Teratomas show various patterns of somatic differentiation and can be divided into mature subtypes, characterized by well-differentiated tissue, and immature subtypes, characterized by incomplete differentiation. They tend to have both low mitotic and apoptotic indices [11]. On occasion, mature teratomas undergo malignant transformation of differentiated tissue, requiring treatment regimens for that specific malignant tissue [12]. Yolk sac tumors and choriocarcinomas display morphologies resembling extra-embryonically differentiated tissue. The former derive from endodermal sinus and secrete α-fetoprotein (AFP) [8], while the latter are composed of cytotrophoblastic and syncytiotrophoblastic cells that typically express β-hCG, as shown in Table 2 [8,12]. ECs are uncommon among MOGCTs but quite frequent among testicular GCTs (TGCTs) [11]. They display features of primitive epithelial cells during the early stages of embryonic development. ECs have the highest mitotic and apoptotic indices of all GCT histopathologic subtypes [11,13]. Furthermore, in more than 50% of cases they express high levels of AFP, β-hCG and LDH [8].

In general, non-dysgerminomas are cisplatin (CDDP)-sensitive and are treated by a combination of surgery and chemotherapy, whereas teratomas are relatively chemo-resistant. In the majority of patients, GCTs consist of a mixture of non-dysgerminomatous histologic components, although a combination of dysgerminoma and non-dysgerminoma components is also possible. Tumor markers may thus vary between histotypes.

Dysgerminomas are most likely to be localized in the ovaries at diagnosis and are often early-stage tumors [14]. However, precise data on the incidence of these tumors by stage at diagnosis is not available due to the rarity of the disease. In women <20 years of age, GCTs account for 58% of all ovarian tumors and should always be suspected in young females with a solid ovarian mass [15,16]. The first choice of treatment is surgery, which, depending on the age and prognosis of the patient should attempt to preserve fertility. There is evidence to suggest that stage IA pure dysgerminoma should be treated with surgery alone because of the low rate of recurrence and also because patients can be successfully treated in the event of relapse [17]. Similarly, patients with stage IA grade 1 immature teratoma do not require adjuvant treatment after adequate surgery [18]. Other data propose managing all-grade immature teratoma and all-stage I dysgerminoma with close surveillance, only treating in the event of relapse [17,19]. A multimodality approach regardless of stage IA disease comprising surgery and platinum-based chemotherapy with bleomycin, etoposide and cisplatin (BEP) has obtained 5-year survival rates of up to 100% for dysgerminomas and 85% for non-dysgerminomatous MOGCTs [20,21,22]. Thus, given its efficacy and tolerability, BEP has become standard adjuvant therapy for women with MOGCTs [19].

Patients with de novo advanced disease require a surgical approach, and a careful balance is needed between achieving optimal cytoreduction and preventing delays in postoperative chemotherapy [19]. The majority of patients with MOGCTs relapse within the first 2 years after surgery and, given the lack of randomized controlled trials, are managed with same therapeutic strategy used for relapsed TGCTs [23]. In fact, BEP has proven effective in prolonging survival in patients with metastatic or recurrent OGCTs, obtaining remission rates of between 75% and 90% [24,25]. Despite this high response rate, there is still a proportion of patients (30–35%) who fail to respond completely or who relapse after completion of first-line chemotherapy. Among these, a fraction will have platinum-refractory disease, defined as radiologic or serologic progression within 4 weeks of prior platinum-based regimen. There are limited data on the efficacy and feasibility of specific chemotherapeutic regimens for this subgroup and prognosis remains very poor.

In the present review we provide an overview of current options and future prospects for the management of female patients with advanced GCTs, focusing on those who are not cured after first-line chemotherapy and are thus candidates for salvage treatment.

## 2. Prognostic Factors

Prognostic factors are an important element in the management of MOGCT because they can help to identify the patients who need more intensive therapeutic strategies. Despite the common origin of GCTs from primordial germ cells, prognostic factors for MOGCT differ from those of TGCTs. There is a well known prognostic classification system for male GCTs incorporating stage, histology and serum biomarkers [26], whereas there are no established prognostic parameters for MOGCTs. Bower et al. reported that OGCT patients <30 years of age treated with chemotherapy showed an improved 3-year survival [27]. However, the importance of their findings was limited by the small number of cases included in the study. A retrospective analysis by Murugaesu et al. demonstrated that initial-stage disease and elevation of both β–hCG and AFP were independent factors of poor prognosis, but that age at diagnosis was not [28]. Univariate and multivariate analyses in a retrospective study carried out by Mangili et al. showed that patient age (>45 years) and treatment in a non referral center were the most important predictors of recurrence in, whereas stage >I and yolk sac histology were independent indicators of poor prognosis [29].

A modified version of the male International Germ Cell Cancer Collaboration Group (IGCCCG) risk classification revealed that patients classified as poor risk on the basis of pre-operative and pre-chemotherapy markers had poorer progression-free (PFS) and overall survival (OS) rates [30]. However, this staging system was not able to distinguish between good and intermediate-risk patients, probably because of the small number of patients in the two groups. Another retrospective study of 42 pediatric cases of germ cell and sex cord-stromal ovarian tumors demonstrated that tumor size and histologic type were not significantly correlated with survival [31]. Furthermore, the level of AFP at diagnosis was not significantly associated with survival or recurrence. A recent study focused on patients diagnosed with advanced stage (II–IV) MOGCT submitted to primary cytoreductive surgery [32]. The authors reported no difference in OS following stratification by histology for dysgerminoma or non-dysgerminoma. Furthermore, the presence of macroscopic residual disease following primary cancer-directed surgery did not correlate with poorer prognosis. Further studies are needed to evaluate the prognostic impact of vital residual disease, as already done for male GCTs [33].

Therefore, as shown above, currently there is not a validated prognostic classification that could help physicians in their clinical practice and further studies are requested [34]. A prognostic classification is also lacking for relapsed/refractory female GCTs. A recent large cooperative study of the International Prognostic Factors Study Group identified prognostic factors for male GCTs at first relapse capable of substantially differentiating prognosis on the basis of the prognostic score (OS rates ranged from 10–70%) [35]. A similar study among several cooperative groups is ongoing to better characterize prognostic factors and the efficacy of second-line therapies in this setting.

## 3. CDDP-Acquired Resistance

When indicated, CDDP-based chemotherapy can cure the majority of patients with GCTs. Conversely, CDDP-refractoriness is associated with aggressive disease and extremely poor prognosis. The molecular basis for this sensitivity or resistance, probably multifactorial, is still poorly understood. CDDP sensitivity appears to correlate with the frequent presence of wild-type TP53, low levels of p53 and the p53 negative feedback regulator, MDM2 (mouse double minute 2 homolog). Moreover, high levels of Oct4 and low levels of cytoplasmatic p21 contribute to the high sensitivity of GCTs to CDDP-based chemotherapy. Thus, the development of CDDP resistance is mediated through altered levels of various key factors [36]. Other mechanisms of CDDP-acquired resistance include reduced drug accumulation, increased detoxification of CDDP in cellular cytoplasm and decreased access of CDDP to DNA inside the nucleus [37]. Although numerous studies have analyzed the mechanisms involved in CDDP resistance in TGCTs, relatively little is known about such mechanisms in OGCTs. Given their common origin, they could be similar. The main mechanisms are as follows.

### 3.1. Role of p53

CDDP sensitivity in GCTs is largely related to the role of p53 and to DNA damage response. In numerous tumor types, CDDP resistance correlates with p53 inactivation at the genetic or protein level given that tumor protein p53 (TP53) is one of the most widely mutated genes in human cancer. Surprisingly, CDDP resistance has been directly linked to TP53 mutations in only a subset of refractory TGCTs, [38]. Thus, mutations in components regulating the p53 pathway are frequent and play an important role in CDDP resistance in TGCTs. Elevated expression of the p53, MDM2 and p21, and reduced expression of octamer-binding transcription factor 4 (Oct-4) and Noxa (a target gene of p53) have been detected in intrinsic CDDP-resistant TGCTs cells prior to CDDP treatment [39,40,41,42,43,44]. p21 is activated by p53 and high expression of the former correlates with the intrinsic CDDP-resistant in TGCTs and may be involved in apoptosis inhibition [41,44,45]. MDM2 is an important antagonist of p53 and increased expression has been detected in CDDP-resistant with respect to CDDP-sensitive TGCT cells [44,45,46].

### 3.2. DNA Methylation

More undifferentiated GCTs such as seminomas, which show higher sensitivity to CDDP, are hypomethylated, whereas non-seminomas have more highly methylated DNA [47]. Furthermore, different methylation profiles have been observed in sensitive and resistant nonseminomas. Koul et al. suggested that promoter hypermethylation of *RASSF1A* and *HIC1* genes are associated with CDDP resistance, while a high incidence of *MGMT* and *RARB* promoter hypermethylation may play a role in the sensitivity of GCTs to CDDP [48]. A number of studies analyzed the effect of demethylating agents such as 5-azacytidine, 5-aza-deoxycytidine and guadecitabine in GCT cell lines, hypothesizing a correlation between global methylation status and response to chemotherapy. Further studies are needed to increase our knowledge about these promising agents [47,49,50,51].

### 3.3. PDGFRb/PI3K/p-AKT Pathway

Deregulation of the PDGFRb/PI3K/p-AKT pathway plays a key role in CDDP resistance [41,52,53] and is associated with the inactivation of PTEN, which also increases the mammalian target of rapamycin signaling. However, Mego et al. failed to demonstrate the efficacy of everolimus (mammalian target of rapamycin inhibitor) against heavily pretreated refractory GCTs [54]. Koster et al. found that high expression of p21 CDDP-resistant EC cells correlated with reduced levels of Oct4 protein and miR-106b seed family members [55]. Conversely, the same authors reported high levels of these miRs and Oct4 in the EC component of chemosensitive GCT patients [41]. PI3K/Akt is needed for the transfer of p21 from the nucleus to the cytoplasm to prevent CDDP-induced apoptosis. Thus, targeting cytoplasmic p21 through PI3K/Akt inhibition sensitizes cells to CDDP-induced apoptosis [55].

### 3.4. Cellular Differentiation

ECs represent an undifferentiated type of tumor that shares expression of pluripotency factors with normal embryonic stem cells. Yolk-sac tumors, choriocarcinomas and teratomas are believed to be derived from more highly differentiated cells [56]. The induction of cellular differentiation could be a way of circumventing CDDP resistance. In fact, cells have a different propensity to undergo apoptosis, probably due to a reduction in proliferation and an inhibition of cell-cycle progression, a phenomenon reported after CDDP-induced DNA damage [57,58]. Abada et al. hypothesized a link between differentiation and resistance to apoptosis in both nonmalignant primordial germ cells and neoplastic germ cells [59]. Interestingly, this has not been seen in other neoplastic diseases. The authors showed that CDDP could induce acute resistance to itself through a differentiation response in pluripotent germ cell tumor cells. Timmer-Bosscha et al. demonstrated that the use of all-trans-retinoic acid (RA) caused differentiation in CDDP-resistant GCTs but reduced apoptotic susceptibility [60]. Conversely, docosahexaenoic acid (DHA) potentiated CDDP-induced cytotoxicity and apoptosis in vitro.

## 4. Conventional Salvage Dose Regimens

Recurrent OGCT is typically detected by an increase in serum tumor markers (β-hCG, AFP and LDH) or by the appearance of new lesions at routine surveillance imaging. Patients who progress after first-line chemotherapy are commonly treated with ifosfamide and CDDP-based regimens, a strategy also used in men with relapsed GCTs [23,61]. For those with residual disease, treatment with paclitaxel, ifosfamide and CDDP (TIP) is recommended as second-line therapy [62] following the high complete response (CR) rate (70%) and low relapse rate observed in TGCTs [63]. In a phase 2 study of the Memorial Sloan Kettering Cancer Center (MSKCC), 46 patients with relapsed TGCTs were treated with TIP as second-line therapy [63]. There was a selection bias for a relatively favorable prognosis, the trial excluding patients with extragonadal GCT (generally considered to have a poor prognosis) and including only patients who obtained a CR to front-line chemotherapy and were CDDP-sensitive, thus with a better prognosis. The paclitaxel dose, given on day 1 as a 24-h infusion, was increased until the maximum tolerated dose of 250 mg/m^2^ was reached; ifosfamide 1500 mg/m^2^ was administered by infusion over 60 min on days 2 to 5; and CDDP 25 mg/m^2^ was infused over 30 min on days 2 to 5. All patients received prophylactic granulocyte colony-stimulating factor (G-CSF). Thirty-two (70%) of the 46 patients obtained a CR and 29 (63%) were disease-free at a median follow-up of 69 months. The 2-year progression-free survival rate was 65%. Fourteen CR patients showed a late relapse and seven achieved a CR to chemotherapy followed by surgery. All seven were disease-free at a median follow-up of 51 months. A British Medical Research Council multicenter phase II trial evaluated the use of second-line TIP based on four courses of paclitaxel 175 mg/m^2^ on day 1 followed by ifosfamide 1 g/m^2^ and CDDP 20 mg/m^2^ on days 1–5 at 3-weekly intervals [64]. Thus, lower doses of chemotherapy were administered with respect to the MSKCC TIP schedule, i.e., −30% paclitaxel, −16.7% ifosfamide and the same doses of cisplatin. In this study patients did not receive G-CSF support after initial BEP chemotherapy. Forty-three patients were enrolled, 26 (60%) of who achieved a CR with negative markers. One-year survival was 70% and failure-free survival was 36%. According to MSKCC risk group, CR was 73% in the group of 26 patients with “good-risk” disease and 41% for the 17 “poor-risk” patients. These results are inferior to those of the MSKCC study where TIP therapy was administered more intensively, at a higher dose, and with G-CSF support, supporting the hypothesis that the higher doses of chemotherapy in the TIP regimen may have had an impact on treatment efficacy. In gynecologic oncology units, the TIP regimen used for cervical cancer included three courses of paclitaxel 175 mg/m^2^ plus ifosfamide 5 g/m^2^ infused over 24 h and CDDP 75 mg/m^2^ every 3 weeks [65]. This regimen is sometimes also used for female GCT. However, compared to MSKCC, the TIP regimen used much lower doses of chemotherapy than that of MSKCC, i.e., −30% paclitaxel, −16.7% ifosfamide and −25% CDDP, without G-CSF support. In light of this, the MSKCC TIP regimen should be the preferred treatment for female GCTs (as it showed the highest best efficacy in male GCT).

CDDP-resistant female GCT is so rare that no prospective studies have ever been conducted in this patient population. The only phase II study performed to date focused on patients with recurrent or advanced dysgerminoma who were CDDP-sensitive and treated with the PVB (CDDP, vinblastine, bleomycin) regimen. However, the trial was closed early because of poor accrual [66]. A retrospective review on MOGCT reported long term-survival in only 10% of patients treated with standard-dose salvage chemotherapy [28]. In male CDDP-resistant GCT, oxaliplatin, gemcitabine and paclitaxel represent the drugs of choice. In a phase 2 trial conducted on 18 male patients with CDDP-refractory non-seminomatous GCT treated with oxaliplatin and gemcitabine, 3 (17%) achieved a prolonged complete remission lasting 44, 20 and 18 months, one of whom underwent surgical resection of residual masses, obtaining a disease-free status [67]. Female patients resistant to CDDP-based treatment can also be treated with vincristine/actinomycin D/cyclophosphamide or paclitaxel/gemcitabine as salvage therapy [19], but prognosis remains poor and worse than in male GCT [34]. Within this context, obtaining a response is an important objective as it may permit surgical intervention, which would increase the chances of long-term survival [68]. No studies investigating the use of an oxaliplatin-gemcitabine regimen in female patients with GCTs have been performed to date and are warranted.

## 5. High-Dose Salvage Chemotherapy

High-dose chemotherapy (HDC) with peripheral blood progenitor cell (PBPC) support is considered an option for salvage treatment in male patients with relapsed GCT [69,70]. Multicycle HDC regimens are currently considered the best option in male GCTs and two regimens are currently used in clinical practice for female GCTs, the Indiana University regimen and the MSKCC regimen [71,72]. The former consists of a mobilizing course with G-CSFs alone or 1–2 cycles of PEI (CDDP, etoposide and ifosfamide) according to the patient’s clinical conditions (chemotherapy is used for bulky and aggressive disease requiring an urgent therapeutic approach) followed by two courses of HDC (tandem regimen) comprising carboplatin 700 mg/m^2^ and etoposide 750 mg/m^2^ daily for 3 days, followed by PBPC infusion [71]. The MSKCC regimen consists of mobilizing chemotherapy with ifosfamide and paclitaxel followed by three HDC courses of carboplatin AUC 7–8 and etoposide 400 mg/m^2^ daily for 3 days, followed by PBPC infusion [72]. For patients with recurrent testicular cancer, HDC can be considered superior to conventional-dose chemotherapy following the results of a multicenter retrospective analysis [69]. A few small studies have evaluated the use of salvage HDC and hematopoietic stem cell transplantation in female patients with relapsed/refractory GCTs. The European Society for Blood and Marrow Transplantation (EBMT) carried out a retrospective analysis of female patients with GCTs treated with salvage HDC [61]. Of the 51 assessable patients, 17 achieved a CR, eight a marker-negative partial remission and five stable disease (SD). There were three treatment-related deaths. 42% of patients were progression-free following HDC at a median follow-up of 87 months [61]. Ammakkanavar et al. treated 13 patients with recurrent malignant OCGTs with the Indiana University regimen. Seven patients achieved a CR, of who four remained disease-free 12, 22, 120 and 270 months after treatment [25].

## 6. New Drugs

Over the last decade several targeted therapies for male GCTs have been evaluated but proven ineffective [73]. Such data are lacking for female patients because female GCTs were not included in these studies. An ongoing phase 2 trial (NCT02533765) is investigating the efficacy of olaparib in GCT patients (male and female) who progressed during CDDP-based regimen or progressed/relapsed after HDC or after at least two different CDDP-based chemotherapy regimens. Olaparib is an oral poly (ADP-ribose) polymerase (PARP) inhibitor. PARP inhibition is a promising therapeutic strategy for cancers characterized by specific DNA defects, in particular neoplastic cells that show a BRCA1 or BRCA2 mutation and are rendered deficient in homologous recombination repair (HR). Tumors with HR deficiency (HRD) cannot repair DNA damage, which may lead to cell death. PARP inhibition eliminates an alternative DNA repair pathway, thus promoting tumor cell death [74,75]. The biologic rationale for the study was provided by other authors who demonstrated that PARP was overexpressed in TGCTs with respect to normal testicular tissue. Mego et al. reported that PARP overexpression was an early event in male GCT development [76]. Patients with low PARP expression in primary GCTs had a better, albeit non-significant, OS than those with high PARP expression (5-year OS 89.2% vs. 78.7%; HR = 0.50, 95% CI 0.21–1.17, *p* = 0.12) [76].

In GCT tumorigenesis, aberrations of the retinoblastoma (RB) pathway are a central event [77], especially in CDDP-resistant tumors [78]. Teratomas have been shown to demonstrate high RB protein (pRB) expression [79,80]. Palbociclib is an oral inhibitor of pRB phosphorylation through the inhibition of cyclin-dependent kinases 4 and 6. Within this context, Vaughn et al. performed a single-arm phase 2 trial of palbociclib in patients with pRB-expressing refractory metastatic GCT. The 24-week PFS rate was 28% and patients with teratoma and teratoma with malignant transformation showed significantly better PFS [81].

The role of immune check-point inhibitors should also be analyzed in these rare tumors. High PD-L1 expression has been reported in TGCTs, indicating that patients could potentially benefit from immunotherapy approaches with programmed death 1 (PD-1) or PD-ligand 1 (PD-L1) inhibition [82]. Furthermore, PD-L1 expression appears to have a prognostic value in patients with GCTs, suggesting that those with high PD-L1 expression are more likely to have (more) aggressive disease and worse survival [83]. However, preliminary results from two studies of pembrolizumab and avelumab in male GCTs did not show significant results [84,85]. A single-arm phase II trial investigated pembrolizumab 200 mg i.v. q3weeks in patients with relapsed GCT who progressed after first-line CDDP-based chemotherapy and after at least one salvage regimen [84]. Twelve patients were enrolled and all had non-seminoma, one of extragonadal origin. There were no partial or complete responses. Radiographic stable disease of 19 and 28 weeks was observed in two patients, both of who had negative PD-L1 staining. In another phase II study, Mego et al. treated eight patients with multiple relapsed and/or refractory GCTs [85], with 10 mg/kg avelumab administered biweekly. Five of the eight patients had CDDP-refractory disease. All had progressed at a median follow-up of 2.6 months. Curiously, no severe immune-related adverse events were reported in either study, probably due to the short duration of exposure to the drugs.

Further studies based on patient selection and specific biomarkers are needed, including, where possible, female GCTs [86]. The main completed and ongoing studies are summarized in Table 3. The recruitment of female patients with GCTs in prospective clinical trials on salvage treatments for male patients is warranted to give female patients the opportunity of potentially benefitting intensive chemotherapeutic approaches and/or new agents.

## 7. Conclusions

Although female GCTs represent an extremely rare disease, the majority of patients can be cured with surgery or CDDP-based treatment. For this reason, the goal of treatments must also be to preserve fertility and reduce the risk of late adverse effects to guarantee good quality of life. Salvage therapy for MOGCT remains an unmet need, with current treatments based mainly on the results of retrospective studies. Several new drugs for this setting have been investigated in recent years, but with disappointing effects.

The present review suggests that HDC is a valid therapeutic option for CDDP-resistant female GCT. However, it is clear that more effective treatments are needed in this subset of patients. In two prospective clinical trials and in major consecutive series in GCTs accepting both genders, female GCT patients represented 3–7% of the overall recruited population [72,87]. In the pediatric population where GCTs represent one of the most common solid tumors and the ovary is a frequent primary site, it is common to enroll both male and female patients in clinical trials. Two important studies in this setting reported that female patients varied from 40% to 70% of the total GCTs [88,89].

On the basis of the rarity of the disease, new clinical trials on GCTs should include both male and female patients to provide prospective data on intensified therapeutic strategies such as HDC and/or new drugs, in particular molecular targeted therapies, for female GCTs. In this respect, the role of cooperative groups at national and international level will be crucial. Moreover, collaborative studies could be performed to validate prognostic classification systems to identify patients who are more likely to benefit from salvage HDC than from other SDC regimens. For the moment, patients with CDDP-resistant MOGCTs should undergo HDC or should be included in clinical trials, when possible. Referral of patients to centers with high expertise in this field is essential.

## Figures and Tables

**Table 1:** Malignant ovarian germ cell tumors classification (according to the World Health Organization (WHO) classification of tumors) [5].

Germ Cell Tumors	Tumor Type
Primitive germ cell tumors	Dysgerminoma
	Yolk sac tumor
	Polyvescicular vitelline tumor
	Glandular variant
	Hepatoid variant
	Embryonal carcinoma
	Polyembryoma
	Non-gestational choriocarcinoma
	Mixed germ cell tumor
Biphasic or triphasic teratoma	Immature teratoma
	Mature teratoma

**Table 2:** Germ cell tumors (GCTs) and their tumor markers [8].

GCTs	Markers		
	AFP	β-hCG	LDH
Dysgerminoma	Normal	May be elevated	May be elevated
Yolk sac tumor	Elevated in all cases	Normal	May be elevated
Embryonal carcinoma	May be elevated	May be elevated	Elevated
Non-gestational choriocarcinoma	Normal	Elevated in all cases	Normal
Mature teratoma	Normal	Normal	Normal
Immature teratoma	May be elevated	Normal	Normal
Mixed germ cell tumor	May be elevated	May be elevated	May be elevated

GCTs, germ cell tumors; β-hCG, β-human chorionic gonadotropin; AFP α-fetoprotein; LDH, lactic dehydrogenase.

**Table 3:** Principal studies for treatment of relapsed/refractory GCT.

Type of Regimen	Tumor Type Assessed	Type of Study	Patients (No. of Female Patients)	Therapy (No. of Patients)	Dose Regimen	Response	Treatment-Related Death (No.)	Study	Recruitment Status
HDC	Female gonadal/extragonadal relapsed/refractory GCTs	Retrospective	51 (51)	Carboplatin, Etoposide and cyclophosphamide (21) Carboplatin, etoposide and ifosphamide (14) Carboplatin and etoposide (13) Carboplatin, etoposide and thiotepa (3)	N.A.	15 CR 9 PR (−) 5 PR (+) 5 SD	3	De Giorgi et al. [60]	Completed
	Female recurrent GCTs	Retrospective	13 (13)	Carboplatin and etoposide	Carboplatin 700 mg/m2 and etoposide 750 mg/m^2^ IV daily for 3 consecutive days	7 CR	none	Ammakkanavar et al. [24]	Completed
New therapy	Male and female refractory GCTs	Phase 2	30 (4)	Palbociclib	125 mg daily for 21 days Q28days	0 CR 24-week PFS 28%	none	Vaughn et al. [80]	Completed
	Male relapsed GCTs	Phase 2	12 (0)	Pembrolizumab	200 mg IV Q3weeks	0 CR 0 SD	none	Adra et al. [83]	Completed
	Male refractory GCTs	Phase 2	8 (0)	Avelumab	10 mg/kg Q14days	12-week PFS 0%,	none	Mego et al. [84]	Completed
	Male and female gonadal/extragonadal relapsed/refractory GCTs	Phase 2	18 (0)	Olaparib	300 mg twice daily continuously	N.A.	N.A.	NCT02533765	Active, not recruiting
	Male and female refractory GCTs	2-stage, phase 2	N.A.	ASP1650	Study to establish the recommended phase 2 dose	N.A.	N.A.	NCT03760081	Recruiting
	Male and female gonadal/extragonadal Refractory GCTs	Randomized, 3-stage, phase 2	N.A.	Durvalumab ± tremelimumab	Durvalumab 1500 mg IV, q4 weeks; Tremelimumab 75 mg IV, both on day 1 and q4 weeks	N.A.	N.A.	NCT03081923	Recruiting

GCTs, germ cell tumors; HDC, high dose chemotherapy; CR, complete response; PR (−), partial remission with negative marker; PR (+), partial remission with positive marker; SD, stable disease; PFS, progression-free survival; N.A., not available.
